# Construction of a Lysine Lactylation- and DNA Damage Repair-Related Gene Signature to Predict the Prognosis and Drug Sensitivity of Breast Cancer Patients

**DOI:** 10.3390/ijms27104493

**Published:** 2026-05-17

**Authors:** Liang Zhu, Chenwei Yuan, Yaorong Li, Yuan Feng, Luoqi Liang, Pinxuan Zhu, Wenjin Yin, Jinsong Lu

**Affiliations:** Department of Breast Surgery, Renji Hospital, School of Medicine, Shanghai Jiao Tong University, Shanghai 200000, China; zhuliang0401@163.com (L.Z.); yuanchenwei@renji.com (C.Y.);

**Keywords:** breast cancer, prognosis, lysine lactylation, DNA damage repair, drug sensitivity

## Abstract

Breast cancer is prevalent and deadly, affecting women worldwide. Increasing research suggests that lysine lactylation (KLA) and DNA damage repair (DDR) play critical roles in tumor progression and that KLA and DDR are interconnected, as KLA can modulate DDR protein function, thereby influencing genome stability and drug response, while DDR signaling can reciprocally reshape lactate metabolism and KLA activity. In this study, we developed a novel prognostic gene signature (KLA and DDR index, KLDRI) based on KLA- and DDR-related genes. Model genes (*PGK1*, *MORF4L2*, *RAD54B*, *RPA3*, *CCND2*) were generated via LASSO-Cox regression. Patients were stratified into high- and low-risk groups according to KLDRI, the robust prognostic value of which was demonstrated via survival and validation analyses in the TCGA cohort and the METABRIC and GSE96058 cohorts, respectively. Tumor microenvironment analysis indicated an immunologically suppressed phenotype in high-risk patients, whereas low-risk patients exhibited an immune-inflamed microenvironment. Drug sensitivity analysis indicated reduced sensitivity to multiple chemotherapy and targeted therapy drugs in the high-risk group. Single-cell transcriptomic analysis revealed differential gene expression patterns between risk groups. A prognostic nomogram based on KLDRI was developed to predict overall survival. Furthermore, functional experiments demonstrated that *RPA3* knockdown suppressed cancer cell proliferation and migration, sensitized cells to cisplatin treatment, and reduced global lactylation, which may serve as a novel biomarker and potential therapeutic target. These findings enhance our understanding of the interplay between KLA, DDR, and breast cancer progression, facilitating the development of personalized therapeutic strategies.

## 1. Introduction

Breast cancer is a leading cause of cancer-related morbidity and mortality affecting women worldwide. According to GLOBACAN 2022, it is estimated that breast cancer constitutes 23.8% of all cancer diagnoses and 15.4% of all cancer-related deaths in women globally [1]. Advanced early detection techniques and multidimensional treatment modalities such as neoadjuvant and adjuvant chemotherapy, endocrine therapy, and targeted therapies have led to significant improvements in patient outcomes. However, disease recurrence and treatment resistance remain challenges, which result in diminished prognosis [2]. Novel prognosis biomarkers should be developed and utilized for the risk stratification of breast cancer patients to guide therapeutic strategies and clinical interventions.

The preference for producing energy through aerobic glycolysis in cancer cells is known as the ‘Warburg effect’ [3]. The accumulation of lactate, converted from the end product of glycolysis, contributes to the acidic conditions that can drive tumor growth and invasion [4,5]. Recently, Zhao et al. identified lysine lactylation (KLA), including histone and non-histone lactylation, as a novel post-translational modification involved in gene transcription regulation and metabolic reprogramming [6]. Increased glycolytic activity and the subsequent accumulation of lactate lead to an overall increase in lactylation in tumors, which has been proven to be associated with malignant behaviors such as proliferation and invasion in tumor cells [7,8]. Histone KLA regulates gene transcription via epigenetic mechanisms [9,10,11,12,13]. Through H3K18 lactylation, the ion channel protein Potassium Two Pore Domain Channel Subfamily K Member 1 (KCNK1) promotes breast cancer proliferation, invasion, and metastasis [14]. Non-histone protein also undergoes KLA. Lactylation-enriched proteome reveals that many DNA damage repair (DDR)-related proteins are lactylated in chemo-resistant cells, indicating a crucial role of lactylation in regulating DNA repair [4,15,16,17]. Cells respond to DNA damage through a highly coordinated and dynamic process called the DDR response, an indispensable surveillance network that preserves the integrity and stability of the genome. DDR deficiency leads to increased genomic instability, which in turn drives cancer initiation and development [18]. The mutation or downregulation of key DDR proteins is associated with impaired DDR, higher incidence, and poorer prognosis of cancer. Recent research has provided insights into lactylation of DDR-related proteins. MRE11 is a key component of the genomic stability maintainer MRE11-RAD50-NBS1 (MRN) complex. MRE11 lactylation has recently been shown to enhance its DNA binding and DNA end resection as well as homologous recombination in breast and ovarian cancer [15]. The lactylation of NBS1, another component of MRN, contributes to a more efficient DDR response and promotes chemotherapy resistance [4]. In addition, lactylation of XLF enhanced non-homologous end joining repair by promoting XLF–Ku80 interaction and recruitment to DNA double-strand break sites [19]. In glioblastoma, Li et al. demonstrated that the highly expressed aldehyde dehydrogenase 1 family member A3 interacts with pyruvate kinase M2 (PKM2). Consequently, PKM2 tetramerization and activation promote lactate production, leading to the lactylation and nuclear translocation of XRCC1 for DDR and therapeutic resistance [16]. Although recent studies have highlighted lactylation as a novel metabolic–epigenetic modification in cancer, its involvement in the DDR process has not been comprehensively investigated.

On these premises, a systematic investigation has yet to be conducted into the impact of KLA and DDR on clinical outcomes in breast cancer patients. In this study, we innovatively analyzed the intricate association between KLA and DDR in breast cancer. Univariate and Least Absolute Shrinkage and Selection Operator-Cox (LASSO-Cox) regression was utilized to identify the model genes most related to prognosis. We clustered breast cancer patients into two risk groups according to five prognostic KLA-DDR-related genes in the TCGA dataset. The prognostic value was further validated on the METABRIC and GSE96058 datasets. In this study, we unveiled a distinguished gene expression signature that can serve as a prognostic indicator for breast cancer patients. We also demonstrated that the knockdown of model gene *RPA3* suppressed cancer cell proliferation and migration and sensitized cells to cisplatin treatment, accompanied by a reduction in global lactylation. Thus, *RPA3* expression may serve as a potential biomarker.

## 2. Results

### 2.1. Identification of Prognostic KLA-DDR-DEGs

**Figure 1:**
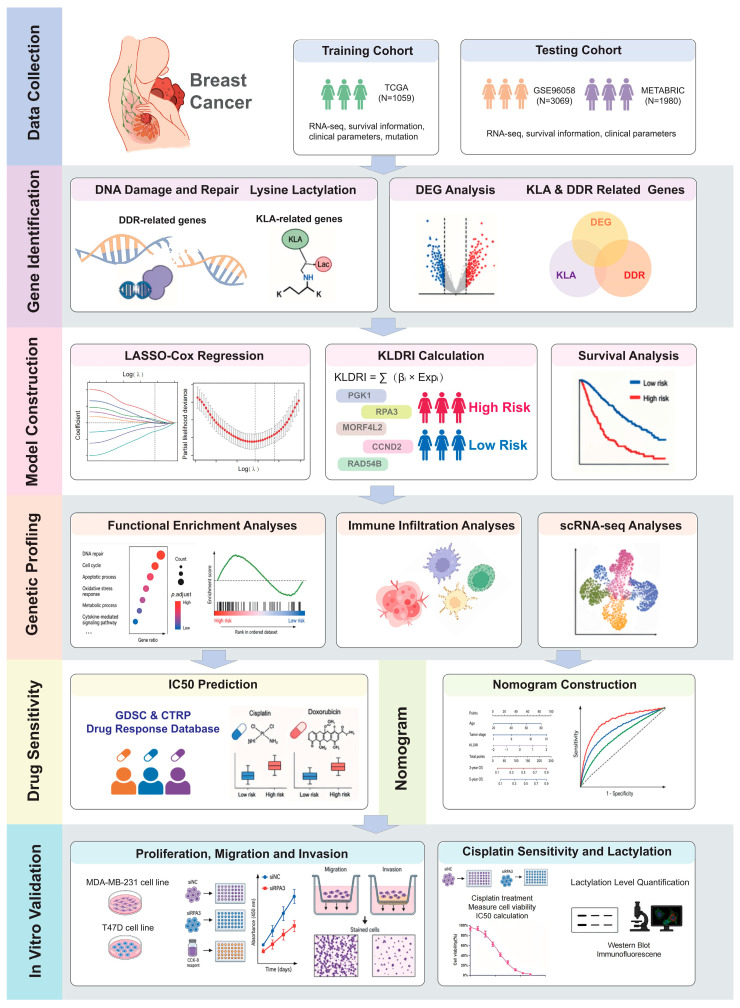
Workflow of the study. Patient and sample data were collected from TCGA, METABRIC, and GSE96058. Differential expression analysis was performed to identify genes associated with KLA and DDR. Then, a LASSO-Cox regression model was applied to screen key prognostic genes and construct KLDRI. KLDRI divided patients into high-risk and low-risk groups, and survival analyses were performed in the TCGA cohort and further validated in METABRIC and GSE96058 cohorts. Functional enrichment analysis, immune cell deconvolution algorithms, and ScRNA seq analyses were utilized to reveal transcriptional and immune cell infiltration landscape. Drug sensitivity predictions were performed for two risk groups. A nomogram integrating clinical features and KLDRI was developed to estimate survival. In vitro experiments were performed to validate the function of the hub gene *RPA3*.

**Figure 2:**
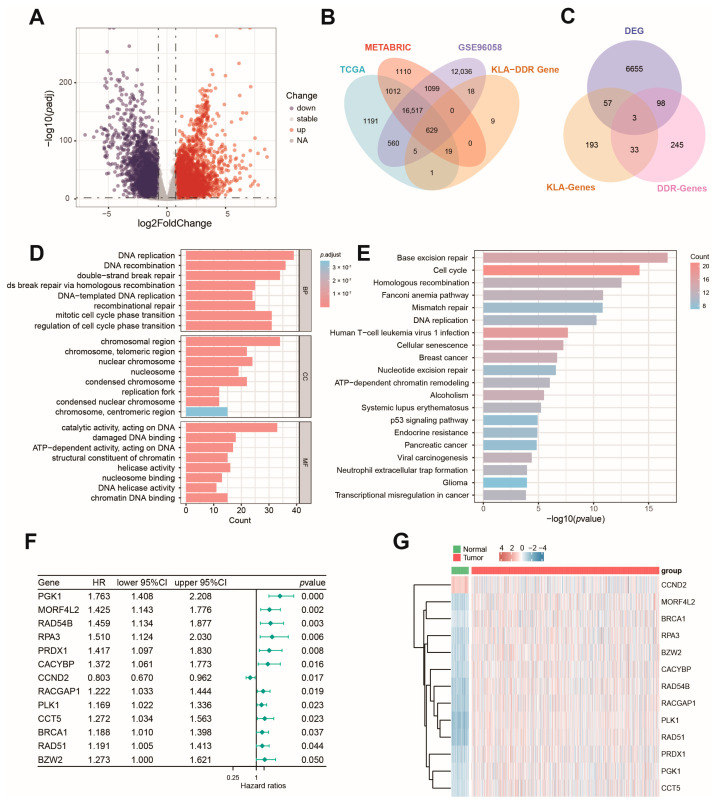
Identification of OS-related DEGs in KLA- and DDR-related genes. (**A**) Volcano plot showing differentially expressed genes between tumor and normal samples in the TCGA cohort. (**B**) Venn diagram illustrating the common KLA-related genes and DDR-related genes in three datasets. (**C**) Venn diagram illustrating the intersection of DEGs and the target gene set. (**D**,**E**) GO (**D**) and KEGG (**E**) enrichment analysis of differentially expressed genes. (BP: Biological Process, CC: Cellular Component, MF: Molecular Function.) (**F**) Univariate Cox analysis selecting 13 OS-related KLA-DDR-DEGs. (**G**) Heatmap showing 13 differentially expressed OS-related KLA-DDR-DEGs.

Figure 1 illustrates the overall design of the study. In the TCGA-BRCA cohort, 6813 DEGs between tumor and normal samples were filtered (|log2FC| ≥ 0.75, adjusted *p* value < 0.01), in which 4042 genes were up-regulated and 2771 down regulated (Figure 2A). Across the three datasets (TCGA, METABRIC, and GSE96058), 629 out of 681 genes (comprising 384 DDR-related genes and 335 KLA-related genes) were identified as common genes detected in all datasets (Figure 2B). Among them, 158 genes were differentially expressed and defined as KLA-DDR-DEGs (Figure 2C). The GO and KEGG enrichment analysis results of KLA-DDR-DEGs are presented in Figure 2D,E. Univariate Cox regression analysis was carried out, and 13 genes were identified as OS-related KLA-DDR-DEGs with a *p* value less than 0.05 (Figure 2F). Notably, most genes such as PGK1, MORF4L2, RAD54B, RPA3, PRDX1, CACYBP, RACGAP1, PLK1, CCT5, BRCA1, RAD51, and BZW2 emerged as unfavorable prognostic factors, while CCND2 functioned as a protective factor for OS. Each OS-related gene had significantly different expression levels between tumor and normal samples, as depicted in the expression heatmap (Figure 2G).

### 2.2. Construction of a Prognostic KLDRI Signature for Breast Cancer Patients

**Figure 3:**
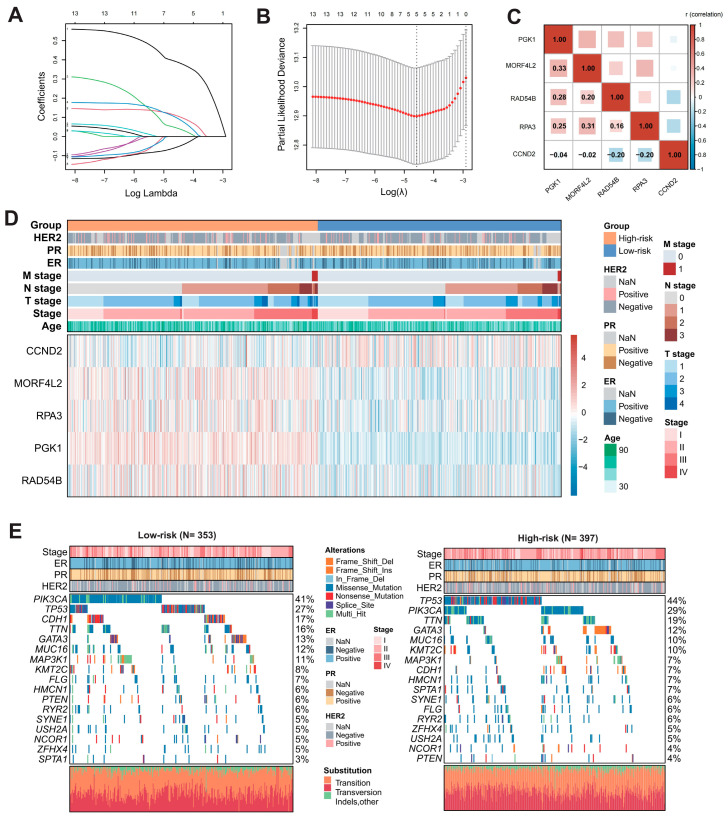
Construction of the KLDRI signature based on the LASSO regression model. (**A**,**B**) Selection of 5 model genes by the LASSO Cox regression model. (**C**) Expression correlation between 5 model genes. (**D**) Expression profile of 5 model genes between high- and low-risk patients. (ER: Estrogen Receptor, PR: Progesterone Receptor, NaN: Undefined) (**E**) Oncoplot of single nucleotide variants in high- and low-risk patients in the TCGA cohort.

LASSO Cox regression analysis identified key prognostic genes. The coefficient profiles of candidate genes decreased toward zero with increasing lambda values (Figure 3A). The optimal lambda value was determined via ten-fold cross-validation based on the minimum criteria (Figure 3B). With a lambda.min value of 5, five KLA-DDR-DEGs were selected: *PGK1*, *MORF4L2*, *RAD54B*, *RPA3*, *CCND2*. A correlation heatmap was generated to illustrate the expression relationships between the five model genes, suggesting potential underlying interactions, particularly for *PGK1* and *MORF4L2* with the highest correlation coefficient (Figure 3C). Based on the LASSO Cox regression result, the risk score for each breast cancer patient was calculated according to the following formula: KLDRI = (0.39010183402774 × *PGK1* exp.) + (0.109159700108414 × *MORF4L2* exp.) + (0.0451856901446805 × *RAD54B* exp.) + (0.0982986506699843 × *RPA3* exp.) + (−0.056211749134365 × *CCND2* exp.). Breast cancer patients in the TCGA cohort were stratified into a high- and low-risk group according to the median KLDRI value. Differences in the gene expression levels of the model genes are displayed in a complex heatmap. Model genes with a hazard ratio (HR) > 1 (*PGK1*, *MORF4L2*, *RAD54B*, *RPA3*) showed higher expression levels in the high-risk group compared to in the low-risk group, whereas *CCND2* (HR < 1) showed lower expression levels in the high-risk group (Figure 3D). Single-nucleotide variation in breast cancer patients between risk groups was evaluated, as illustrated with a mutation waterfall diagram (Figure 3E). The most frequently mutated gene in the low- and high-risk groups was *PIK3CA* (41%) and *TP53* (44%), respectively.

### 2.3. Prognostic Value of KLDRI Signature in Training and Validation Cohorts

**Figure 4:**
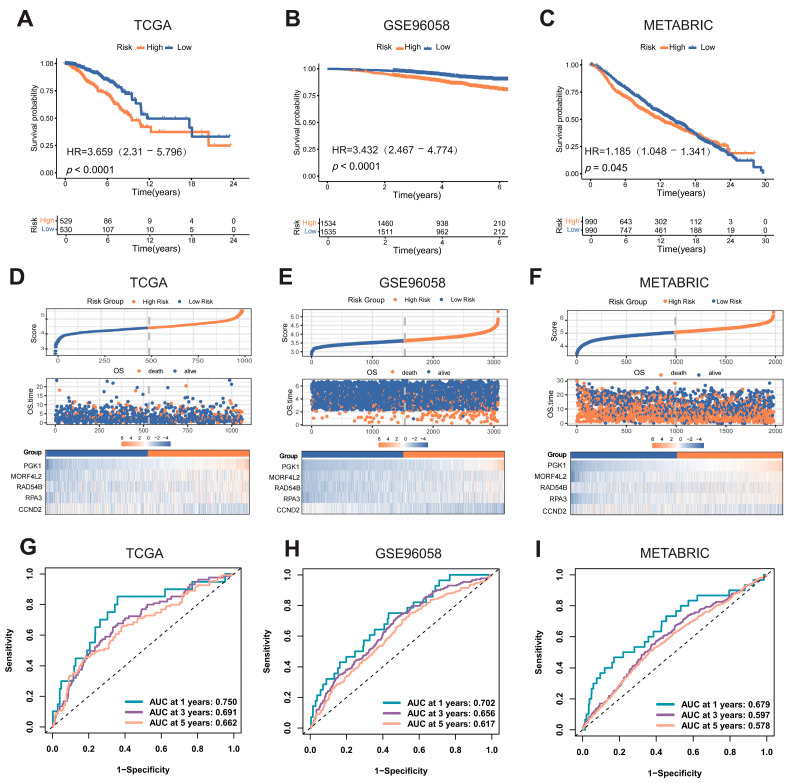
Internal training and external validation of the KLDRI risk model in TCGA, GSE96058, and METABRIC cohorts. (**A**–**C**) Kaplan–Meier curves for high- and low-risk patient groups in TCGA (**A**), GSE96058 (**B**), and METABRIC (**C**) cohorts. (**D**–**F**) Distribution of OS events and differentially expressed model genes between KLDRI risk groups in TCGA (**D**), GSE96058 (**E**), and METABRIC (**F**) cohorts. (**G**–**I**) Receiver operating characteristic (ROC) curves of KLDRI risk group in TCGA (**G**), GSE96058 (**H**), and METABRIC (**I**) cohorts.

We compared differences in survival time based on risk group stratification. For breast cancer patients in the training cohort (TCGA), the Kaplan–Meier curve showed that patients in the high-risk group exhibited poorer prognosis than those in the low-risk group (Figure 4A). Survival analysis results for two validation cohorts (GSE96058, METABRIC) aligned with those for the training cohort (Figure 4B,C). The distribution of OS events, risk scores, and model gene expression in the three cohorts is displayed in Figure 4D–F. Patients were ranked by risk score from low to high. As the risk score increased, the number of OS events also rose (particularly in the GSE cohort), which is consistent with the survival analysis result. The expression of four unfavorable prognostic genes (*PGK1*, *MORF4L2*, *RAD54B*, and *RPA3*) was higher in the high-risk group than in the low-risk group. The ROC curve confirmed the discriminative ability of the KLDRI risk group stratification (Figure 4G–I).

### 2.4. Clinical Relevance of KLDRI Signature

**Figure 5:**
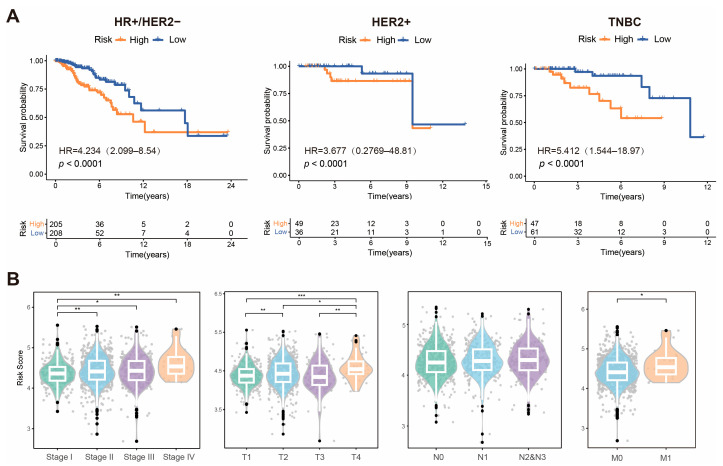
Clinical relevance of the KLDRI signature. (**A**) Kaplan–Meier curves for high- and low-risk patients across the three subtypes in the TCGA cohort. (**B**) Correlation between risk score and tumor stage, T stage, N stage, and M stage. (* *p* < 0.05; ** *p* < 0.01; *** *p* < 0.001).

The prognostic value of KLDRI was also confirmed in different breast cancer subtypes, including hormone receptor positive/HER2 negative (HR+/HER2−), HER2 positive, and triple negative breast cancer (TNBC; Figure 5A). After validating the prognostic role of the KLDRI signature, we evaluated the relevance between KLDRI and clinical characteristics (Figure 5B). Tumor stage, T stage, and M stage were all positively correlated with KLDRI, whereas lymph node metastasis showed a similar trend, but no statistically significant difference.

### 2.5. Identification of DEGs Between Risk Groups and Function Enrichment Analysis

**Figure 6:**
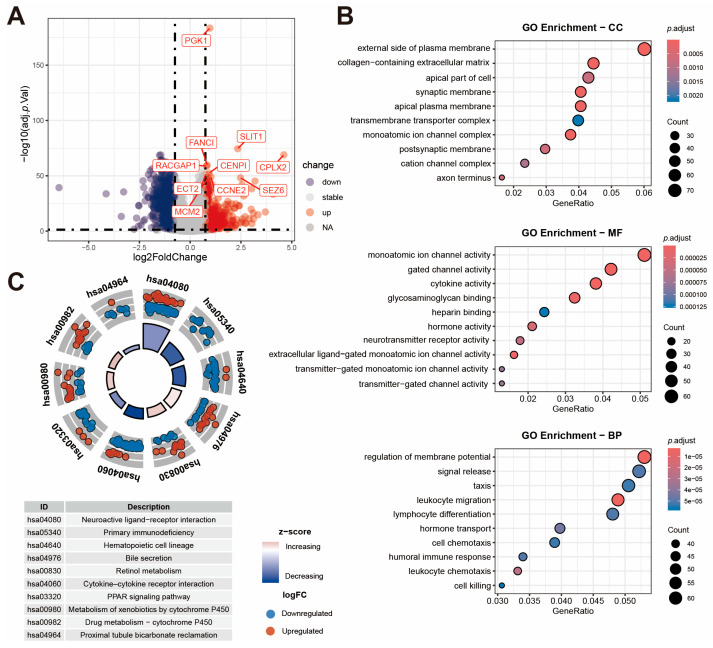
Features of DEGs between high and low KLDRI patients. (**A**) Volcano plot showing differentially expressed genes between high and low KLDRI patients in the TCGA cohort. (**B**) GO enrichment analysis of DEGs. (**C**) KEGG enrichment analysis of DEGs.

To explore the potential molecular mechanism and therapeutic target, we performed DEGs and functional enrichment analyses between the two KLDRI groups. A volcano plot (Figure 6A) shows that 496 upregulated DEGs and 870 downregulated DEGs were identified (|log2FC| ≥ 0.75, *p* < 0.01). GO enrichment analysis (Figure 6B) showed significant enrichment in the external side of the plasma membrane (cellular components, CCs), monoatomic ion channel activity (molecular functions, MFs), and regulation of membrane potential (biological processes, BPs). KEGG enrichment analysis (Figure 6C) revealed restrained cytokine–cytokine receptor interaction in the high-KLDRI group, displaying an immune-suppressive microenvironment. P450-related drug metabolism pathways were found to be enriched in the high KLDRI group and indicated the altered drug metabolic patterns of anti-cancer drugs, potentially contributing to drug resistance. GSEA (Gene Set Enrichment Analysis) revealed that the high-KLDRI group was positively enriched in DNA replication, homologous recombination, and pentose and glucuronate interconversion pathways. In contrast, the low-KLDRI group was positively enriched in JAK-STAT signaling, cytokine–cytokine receptor interaction, and chemokine signaling pathways (Supplementary Figure S1).

### 2.6. Altered Immune Landscape Between KLDRI Groups

**Figure 7:**
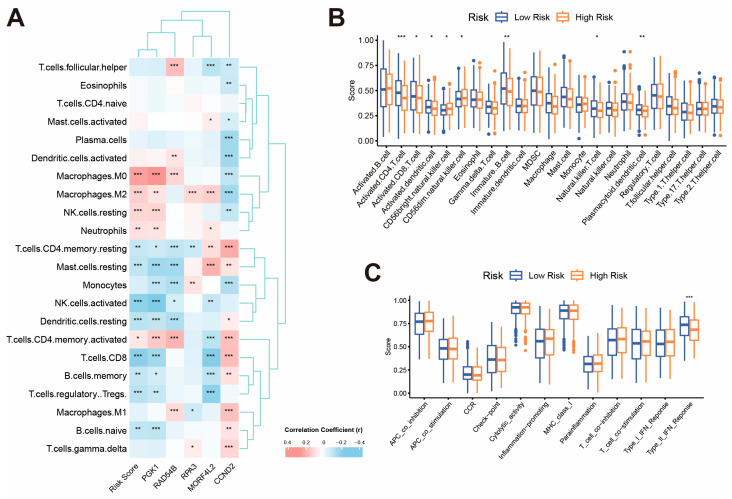
Immune cell infiltration landscape between high-risk and low-risk TCGA patients. (**A**) Correlation between risk score, model genes and 22 immune cells infiltration calculated by CIBERSORT algorithm. (**B**) Analysis of infiltration of 23 immune cells in the high-risk and low-risk groups by ssGSEA. (**C**) Analysis of immune cell function in the high-risk and low-risk groups by ssGSEA. (* *p* < 0.05; ** *p* < 0.01; *** *p* < 0.001).

The tumor microenvironment plays a significant role in facilitating tumor growth, metastasis, and treatment response. We performed multiple analyses to assess the differences in the immune microenvironment between high- and low-risk groups. The CIBERSORT algorithm showed slightly increased M0 and M2 macrophages, and the quantiseq algorithm showed decreased CD4+ and CD8+ T cells in high-risk groups. Correlation analysis, carried out using the CIBERSORT algorithm, revealed that as the risk score increased, the proportion of M0 macrophages, M2 macrophages, resting NK cells, neutrophils and activated CD4+ memory T cells increased, while CD8+ T cells, activated NK cells, resting mast cells, resting dendritic cells, Treg cells and naïve B cells decreased (Figure 7A). We also performed ssGSEA analysis and observed a reduction in various immune cell components in the high-risk group, especially CD4+ T, CD8+ T and dendritic cells (Figure 7B). The type II IFN response was also impaired in the high-risk group (Figure 7C). Supplementary Figure S2 shows a heatmap containing the results of three deconvolution algorithms, displaying the overall distribution of immune cell proportions. Collectively, high KLDRI was shown to be related to an immunosuppressive microenvironment, which might contribute to the poor prognosis of high-KLDRI patients.

### 2.7. Drug Sensitivity Analysis Between KLDRI Groups

**Figure 8:**
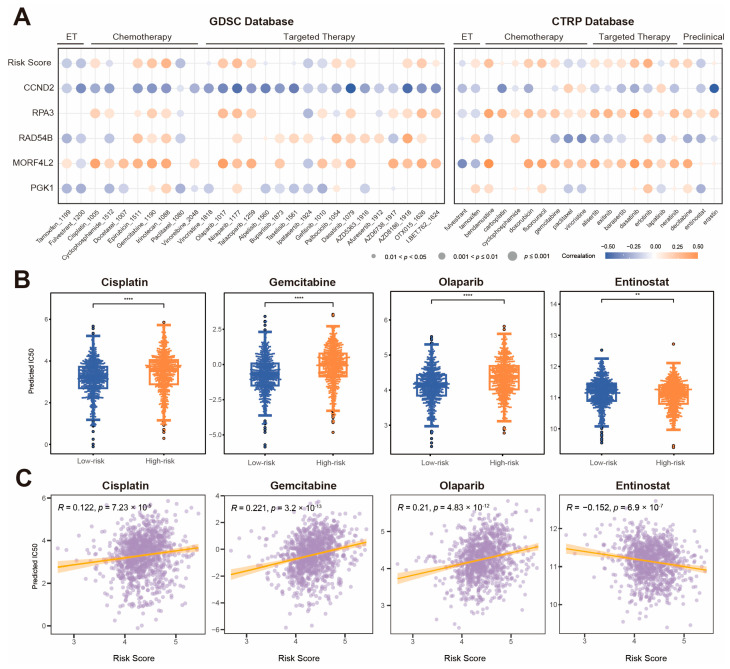
Efficacy of KLDRI signature in predicting drug sensitivity. (**A**) Dot plot of the correlation between drugs, KLDRI risk score and model genes. (**B**) Box plots illustrating predicted IC50 of potential drugs between high-risk and low-risk groups. (**C**) Correlation analysis of the KLDRI risk score and predicted IC50 of potential drugs. (** *p* < 0.01; **** *p* < 0.0001).

To evaluate the treatment response differences between two KLDRI groups, we used the R package ‘oncopredict’ to predict the half maximal inhibitory concentration (IC50) of drugs for breast cancer patients in the TCGA cohort using both the Genomics of Drug Sensitivity in Cancer (GDSC) and Cancer Therapeutics Response Portal (CTRP) databases. Figure 8A illustrates the correlation between KLDRI, five model genes and the predicted IC50 value of each drug. Positive correlations (orange dots) indicate that higher gene expression or higher KLDRI was associated with increased IC50 value, suggesting potential drug resistance, while negative correlations (blue dots) indicate that higher expression or KLDRI was associated with decreased IC 50 value, suggesting greater drug sensitivity. The KLDRI and expression level of adverse prognostic genes (e.g., *MORF4L2*, *RPA3*) were found to be positively correlated with the predicted IC50 of most breast cancer-related drugs, such as cisplatin, gemcitabine, olaparib, and doxorubicin, suggesting drug resistance to these agents, whereas the favorable prognostic gene *CCND2* exhibited the opposite response. Nevertheless, the high-risk group also exhibited increased sensitivity to certain drugs, including fulvestrant, paclitaxel and entinostat. The predicted IC50 of cisplatin, gemcitabine, olaparib, and entinostat is illustrated according to the KLDRI risk score as a categorical (Figure 8B) or continuous variable (Figure 8C). The results show positive correlation between KLDRI and the IC50 of cisplatin, gemcitabine, and olaparib, indicating that KLDRI was associated with reduced drug sensitivity. In contrast, KLDRI was negatively correlated with the IC50 of entinostat, suggesting enhanced sensitivity in high-risk patients.

### 2.8. KLDRI Gene Distribution and Altered Cell Communication in the Microenvironment at the scRNA-Seq Level

**Figure 9:**
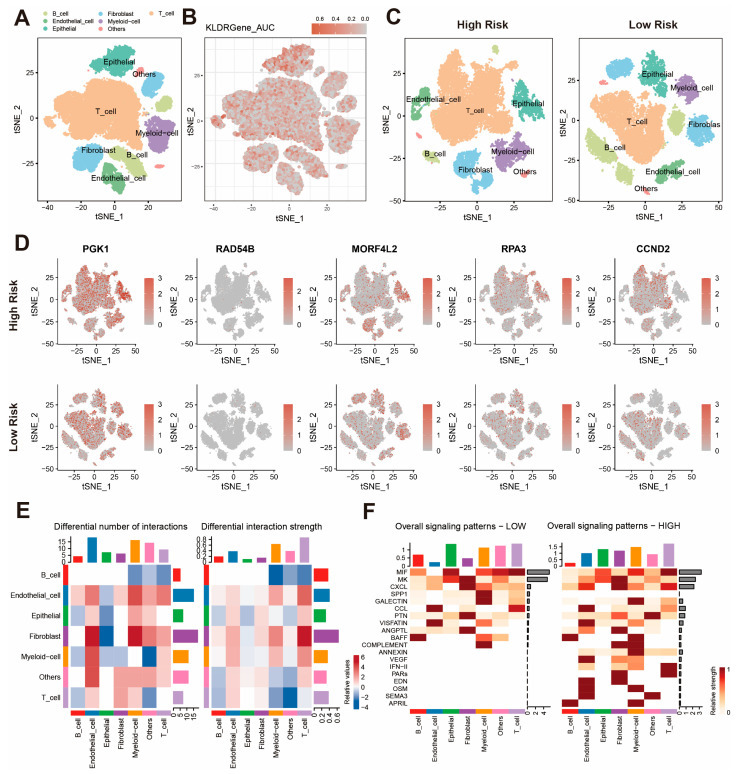
Single-cell RNA seq analysis. (**A**) t-SNE visualization of cell-types in GSE176078. (**B**) t-SNE visualization of AUCell score distribution. (**C**) t-SNE visualization of cell-types between high-risk and low-risk samples. (**D**) Feature plot of KLDRI genes in high-risk and low-risk samples. (**E**) Heatmap of differential cell interactions between two risk groups. (**F**) Heatmap of overall signaling patterns differences between two risk groups.

We performed scRNA-seq analysis based on GSE176078 dataset to further explore the distribution of KLDRI genes across different cell types. The T-distributed stochastic neighbor embedding (t-SNE) visualization (Figure 9A) and AUCell scoring (Figure 9B) show widely distributed KLDRI gene activity across various cell types, with preferential enrichment in epithelial cells. We also calculated the risk score of 26 samples according to the average expression level of model genes and stratified them into two risk groups. The t-SNE visualization of cell types in the two risk groups and the KLDRI gene expression feature are shown in Figure 9C,D. Up-regulated KLDRI gene expression was observed in the high-risk group, especially within epithelial and T cells (Figure 9D). The heatmap of different interactions revealed widespread remodeling of intercellular communication patterns. Compared with the low-risk samples, high-risk samples displayed a generally increased number and intensity of interactions among immune and stromal compartments (Figure 9E). Diverse signaling patterns were also observed between the two risk groups (Figure 9F). These findings imply that high KLDRI may contribute to remodeling of the tumor microenvironment through ligand–receptor interactions, rather than being confined to tumor cells alone.

### 2.9. Establishment of a Nomogram Risk Model

**Figure 10:**
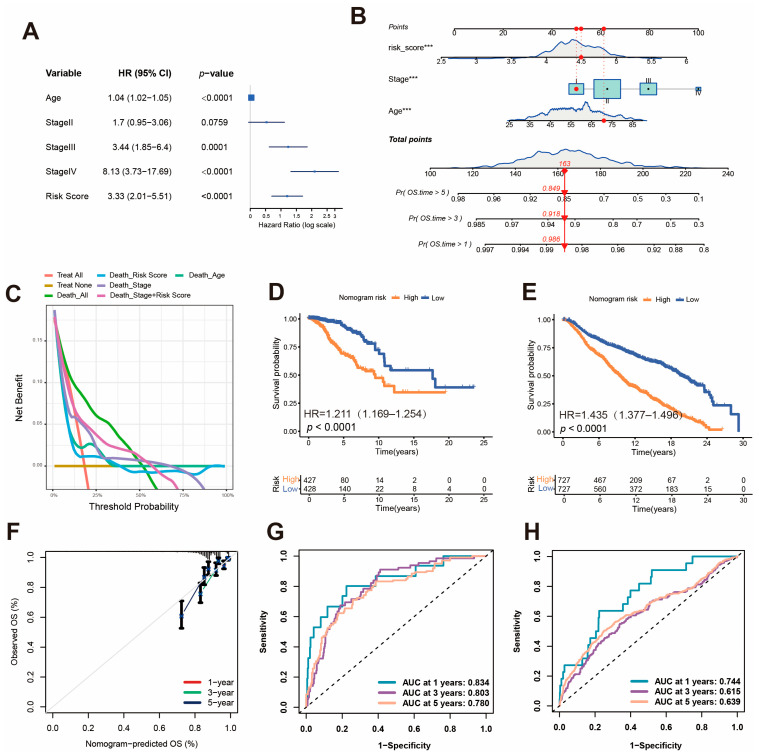
Establishment and validation of the nomogram survival model. (**A**) Multivariate Cox model of risk score, age and stage in TCGA cohort. (**B**) Nomogram for predicting breast cancer patients’ 1-, 3- and 5-year OS probability. (**C**) DCA curve. (**D**,**E**) Kaplan–Meier curves for high and low nomogram risk patient groups in TCGA (**D**) and METABRIC cohort (**E**). (**F**) Calibration curves of the nomogram. (**G**,**H**) ROC curves of nomogram in TCGA (**G**) and METABRIC cohort (**H**). (*** *p* < 0.001).

Multivariate Cox regression analysis was performed to evaluate the prognostic value of KLDRI combined with clinical characteristics, including age and tumor stage. Higher KLDRI, older age and higher tumor stages (stage II, III, and IV compared to stage I) were all risk factors for the survival outcomes of breast cancer patients in the TCGA cohort, as shown in Figure 10A. A nomogram risk model was then established to predict the 1-, 3-, and 5-year survival probability of patients (Figure 10B). DCA demonstrated clinical benefits of models incorporating different variables and confirmed that the nomogram model (KLDRI + Age + Stage) provided the greatest clinical benefit (Figure 10C). A calibration curve showed the accuracy of predicting 1-, 3- and 5-year surviving probability of the nomogram risk model (Figure 10F). Survival analysis (Figure 10D) and ROC curve analysis (Figure 10G) based on nomogram risk group stratification were performed, the results of which indicate that breast cancer patients with higher nomogram risk had a poor prognosis. This result is consistent with that of the validation cohort (METABRIC; Figure 10E,H).

### 2.10. Knockdown of RPA3 Reduces Proliferation and Migration In Vitro

**Figure 11:**
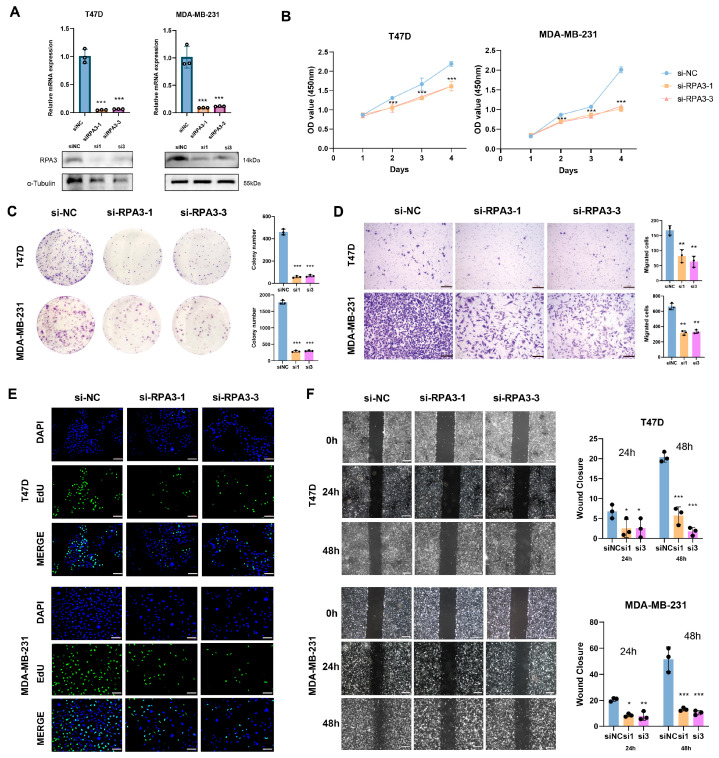
Silencing *RPA3* reduces breast cancer cell proliferation and migration. (**A**) Knockdown efficiency of two siRNA sequences targeting *RPA3* (si-RPA3-1 and si-RPA3-3) in T47D and MDA-MB-231 cells validated by qRT-PCR and Western blot assays. (**B**) CCK8 assay analysis. Cell viability of T47D and MDA-MB-231 cells transfected with siNC, si-RPA3-1 or si-RPA3-3 was measured at day 1, 2, 3 and 4 after transfection. (**C**) Cloning formation analysis. Representative images and quantification showed the number and size of colonies in T47D and MDA-MB-231 cells. (**D**) Transwell experiment analysis. Cells migrated to the lower chamber were imaged to evaluate the migratory ability. (scale bar: 200 μm) (**E**) EdU assay evaluating DNA synthesis and proliferative activity in T47D and MDA-MB-231 cells. Green fluorescent signals represent EdU-positive proliferating cells. Blue fluorescent signals represent DAPI for nuclear staining. (scale bar: 100 μm) (**F**) Scratch assay was performed to evaluate the migratory ability of T47D and MDA-MB-231 cells. Representative images and quantitative analysis of migration rates are shown. (scale bar: 200 μm) (* *p* < 0.05; ** *p* < 0.01; *** *p* < 0.001).

qRT-PCR validation for model genes was carried out in a series of breast cancer cell lines, including T47D, SKBR-3, and MDA-MB-231, using MCF-10A, a normal breast epithelial cell line, as a normal control (Supplementary Figure S3). The results show that model genes implying poorer prognosis were up-regulated to a different extent in breast cancer cells (*PGK1*, *RPA3*, *MORF4L2* and *RAD54B*). Considering the inadequate investigation of RPA3 in breast cancer, a series of in vitro assays was carried out on T47D and MDA-MB-231 cell lines. The knockdown efficiency of siRNA targeting *RPA3* was evaluated via qRT-PCR and Western blot (Figure 11A). A marked reduction in cell viability was observed in siRPA3 groups compared to the control group on days 3 and 4 (Figure 11B). Similarly, colony formation assay revealed a marked reduction in the number of colonies upon *RPA3* suppression (Figure 11C). Moreover, the 5-ethynyl-2′-deoxyuridine (EdU) incorporation assay revealed a decreased proportion of EdU-positive cells after RPA3 knockdown, demonstrating a significant decrease in DNA synthesis, further confirming the inhibitory effect on cell proliferation (Figure 11E). In addition, transwell assays showed decreased cells penetrating the membrane and cell scratch assays showed delayed wound closure rate, suggesting that *RPA3* knockdown attenuated the migratory ability of T47D and MDA-MB-231 cells, indicating its involvement in promoting tumor cell motility (Figure 11D,F). These results collectively suggest that RPA3 plays a critical role in promoting proliferation and metastasis-related phenotypes in breast cancer cells.

### 2.11. Silencing of RPA3 Enhanced Cisplatin Sensitivity and Reduced Global Protein Lactylation

**Figure 12:**
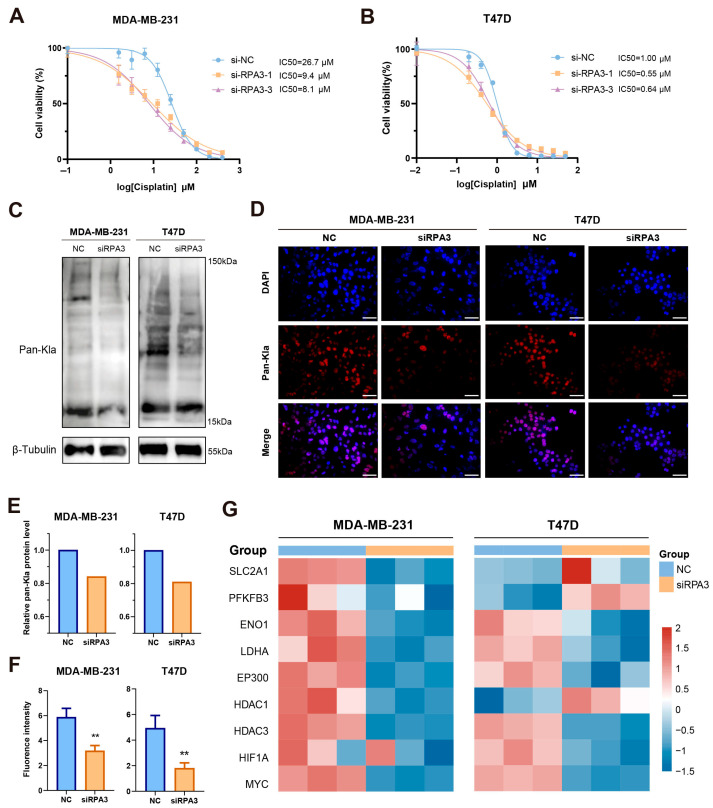
Silencing of *RPA3* enhanced cisplatin sensitivity and reduced global KLA level. (**A**,**B**) Cisplatin IC50 value of MDA-MB-231 and T47D cells transfected with siNC, siRPA3-1, or si-RPA3-3 determined via CCK8 assay. (**C**) Western blot assay for pan-Kla level of T47D and MDA-MB-231 cells following RPA3 knockdown. (**D**) Immunofluorescence assay for pan-Kla level of T47D and MDA-MB-231 cells following RPA3 knockdown. Red fluorescent signals represent Pan-Kla-positive cells. Blue fluorescent signals represent DAPI for nuclear staining. (scale bar: 100 μm) (**E**) Analysis of Western blot assay data. (**F**) Analysis of immunofluorescence assay data. (**G**) Heatmap showing the relative mRNA expression levels of glycolysis- and lactylation-related genes determined via qPCR assay in MDA-MB-231 and T47D cell lines following *RPA3* knockdown. (** *p* < 0.01).

Given the above analyses, we evaluated the sensitivity of breast cancer cells to cisplatin after *RPA3* knockdown. Specifically, *RPA3* knockdown reduced cell viability and increased cytotoxic responses upon cisplatin exposure compared with the negative control in MDA-MB-231 and T47D cells (Figure 12A,B). *RPA3* silencing decreased the IC50 value of cisplatin, indicating enhanced drug sensitivity and the potential contribution of RPA3 to chemotherapy resistance. Moreover, to determine whether *RPA3* silencing affects KLA, we assessed global protein lactylation level (Pan-Kla) via Western blot (Figure 12C,E) and immunofluorescence assay (Figure 12D,F), which demonstrated decreased global lactylation levels following RPA3 knockdown. Consistent with this observation, a qPCR experiment revealed that multiple genes involved in glycolysis and lactylation were downregulated to varying degrees (Figure 12G). Notably, the genes encoding the key lactate metabolic enzyme lactate dehydrogenase A (LDHA) and lactylation ‘writer’ EP300 were both downregulated in the two breast cancer cell lines after *RPA3* knockdown, suggesting a functional linkage between RPA3 and lactylation regulation.

## 3. Discussion

An increasing number of studies have shown that KLA and DDR both play vital roles in breast cancer development and progression. Although previous studies have separately developed prognostic models for KLA and DDR in breast cancer patients [20,21], no research has yet examined their combined effect and delved into the genomic characteristics and tumor microenvironment. In this study, we developed a novel KLA- and DDR-related gene signature (KLDRI) to improve risk stratification in breast cancer. Our findings suggest that integrating these two biological processes may provide a more comprehensive understanding of tumor behavior and patient prognosis.

Accumulating evidence suggests that KLA and DDR not only independently contribute to tumor development but also interact with each other to exacerbate tumor progression and treatment resistance. We constructed KLDRI with the five KLAGs and DDRGs (PGK1, MORF4L2, RAD54B, RPA3, CCND2) that best predict survival outcome. PGK1 is a key enzyme in the glycolytic pathway. Creating a hypoxic and lactate-enriched microenvironment increases the abundance of lactylation modification and promotes tumorigenesis [22,23]. PGK1 itself has been shown to undergo lysine crotonylation and facilitate breast cancer development [24], which suggests that PGK1 contains modifiable lysine residues, providing receptor sites for lactylation. MORF4L2 is a component of the NuA4 histone acetyltransferase complex, involved in chromatin modification, gene regulation, and DNA repair [25]. Recently, it has been reported to induce an immunosuppressive microenvironment in TNBC cells, contributing to chemotherapy resistance [26]. RAD54B is a member of the DNA-repairing protein RAD54 family and participates in homologous recombination repair, thus maintaining genomic stability [27]. High expression of RAD54B promotes tumor growth and predicts poor prognosis in luminal A breast cancer patients [28]. Not much evidence has been accumulated for the role of RPA3 in basic breast cancer research. Evidence from gastric cancer and hepatocellular cancer has indicated that RPA3 is associated with tumorigenesis and poor survival [29]. The specific mechanisms of RPA3 in breast cancer warrant further investigation. CCND2 is a regulator of cyclin-dependent kinase 4 and 6. Qin et al. regarded CCND2 as a risk factor for breast cancer, and its inhibition can attenuate proliferation and progression in MCF-7 cells [30]. Li et al. observed decreased CCND2 expression due to the hypermethylation of its promoter in breast cancer patients, which serves as an independent detrimental factor for prognosis in both tested samples and the public dataset [31]. Although CCND2 had an HR < 1 in the survival analysis of this study, it is likely driven by its strong correlation with ER positivity, a subtype exhibiting a generally favorable prognosis. Functionally, CCND2 remains an oncogenic driver of CDK-mediated cell-cycle progression, predominantly active in ER-positive tumors. Overall, a higher KLDRI score is associated with an unfavorable prognosis and may contribute to therapeutic resistance through multiple mechanisms.

The mutation frequencies in two KLDRI risk groups differed significantly, with the high-risk group showing a higher prevalence of TP53 mutation, indicating stronger tumorigenic biological processes such as an invalidated cell-cycle checkpoint, genomic instability, inhibited apoptosis, and metabolic alterations [32]. Furthermore, p53 undergoes lactylation through AARS1, which is found to be an intracellular lactate sensor that mediates global KLA. The lactylation of p53 by AARS1 at K120 and K139 residues impairs its DNA binding process [33]. Considering the higher glycolytic activity in high-risk group patients, global KLA is more likely to be prevalent and thereby suppress the activity of the residual wild-type p53, contributing to their poor prognosis. KLDRI score was significantly associated with tumor stage, T stage, and M stage, suggesting its potential relevance to tumor progression. However, no significant association was observed between KLDRI and N stage. Although no statistically significant difference was observed, the KLDRI score tended to increase with higher N stage. The relationship between the model and lymph node involvement remains unclear, as it may be influenced by cohort characteristics or sample size, and requires further validation in independent datasets.

The tumor microenvironment plays a vital role in tumor development, progression, and treatment response. Immune deconvolution algorithms also indicated an immunosuppressive microenvironment featured by increased M2 macrophages and decreased CD4/8+ T cells in high-risk group patients. M2 macrophages are considered to play an anti-inflammatory and immunosuppressive role by secreting anti-inflammatory cytokines like IL-10 [34]. Risk factor genes such as PGK1, RAD54B, and MORF4L2 are all positively correlated with the predicted M2 macrophage proportion and negatively correlated with the predicted CD8+ T cell proportion individually, indicating their role in modulating the tumor microenvironment and steering it toward immunosuppression. However, these results were derived from computational methods and require further experimental validation.

According to drug sensitivity analysis, high-risk group patients may respond poorly to multiple chemotherapy and targeted therapy drugs (i.e., cisplatin, gemcitabine, niraparib, erlotinib). Nevertheless, they may have increased sensitivity to certain medications such as fulvestrant, paclitaxel, and entinostat. Additionally, entinostat is an oral histone deacetylase inhibitor (HDACi) that has been investigated in phase II and III clinical trials with promising efficacy in patients resistant to endocrine therapy and cyclin-dependent kinase inhibitors (CDKi) [35]. Despite resistance to many drugs, novel targeted therapies may be the preferred alternatives for patients with high KLDRI.

In this study, we demonstrated that knockdown of RPA3 significantly suppressed breast cancer proliferation and migration as well as enhanced cisplatin sensitivity and decreased global KLA level. RPA3 is the smallest subunit of the heterotrimeric Replication Protein A (RPA) complex, binding to the single-stranded DNA (ssDNA), thereby stabilizing the DNA for repair. This study is the first to demonstrate the role of RPA3 in breast cancer, supported by previous reports in nasopharyngeal carcinoma and lung adenocarcinoma [36,37]. Decreased KLA levels following RPA3 knockdown suggest interplay between RPA3 and lactylation. Given that recent studies have demonstrated that RPA1 undergoes KLA [38], it is conceivable that the lactylation-mediated modulation of RPA1 may alter the assembly, stability, or functional output of the RPA heterotrimer, thereby indirectly affecting RPA3-dependent replication stress responses. Tumors with enhanced lactate production and elevated replication stress may exhibit increased dependency on intact RPA complex function. Under such conditions, targeting RPA3 could sensitize cancer cells to metabolic perturbations or lactylation-associated DDR dysregulation. This suggests potential therapeutic interplay between lactylation-driven metabolic reprogramming and RPA3-targeted DDR intervention. Although no specific inhibitors directly targeting RPA3 are currently available, the RPA complex inhibitor HAMNO ((1Z)-1-[(2-hydroxyanilino)methylidene]naphthalen-2-one) has been developed and shown to be therapeutically effective in squamous cancer [39]. Given the essential role of RPA3 in maintaining genomic stability and facilitating homologous recombination repair, it is by no means inconceivable that the selective inhibition of RPA3 may disrupt DNA repair processes in cancer cells, particularly those already harboring deficiencies in the DNA damage response. Therefore, RPA3 may represent a potentially valuable therapeutic target, and further efforts are warranted to develop RPA3-specific inhibitors. Interestingly, decreased pan-KLA level and expression of several glycolysis- and lactylation-related genes followed by *RPA3* knockdown were observed, suggesting a potential association between RPA3 and lactylation regulation. Given the emerging evidence that KLA participates in tumor progression and therapeutic resistance, these findings further support the rationale for integrating KLA- and DDR-related genes in the present study. However, the precise molecular mechanism linking RPA3 to glycolytic metabolism and lactylation remains to be further investigated.

Although our model has shown good performance in both training and validation cohorts, there remain some limitations of this study. Firstly, this study is based on retrospectively collected public datasets, which are limited by certain biases, making it less reliable for definitive conclusions compared to prospective studies. Prospective validation and real-world clinical cohorts are warranted to further validate the clinical applicability of the KLDRI model. Secondly, the integration of KLDRI is primarily based on correlative analyses. With emerging evidence suggesting biological interplay between KLA and DDR at epigenetic and metabolic levels, future studies integrating multi-omics approaches and functional experiments (e.g., lactylation profiling of DDR proteins) will be necessary to validate and refine the proposed interaction network. Notably, the drug sensitivity analysis in the study was based on in silico analyses and cannot fully represent drug response evaluations in vitro and in vivo. Despite the observed association between RPA3 and KLA level alteration, the precise molecular mechanisms remain to be fully elucidated. As a relatively conserved DDR protein, the function of RPA3 demands complete characterization. Future studies are warranted to determine if RPA3 regulates the transcription of glycolytic enzymes, modulates metabolic flux, or influences the activity of KLA writers and erasers through biochemical and epigenomic approaches. Moreover, in vivo models, targetability assessment, and development of specific inhibitors will be required for determining the therapeutic role of RPA3.

## 4. Materials and Methods

### 4.1. Data Acquisition and Preprocessing

RNA sequencing data, clinical information, and single-nucleotide variant (SNV) information of breast cancer patients were obtained from public databases, including The Cancer Genome Atlas (TCGA, https://portal.gdc.cancer.gov/), Molecular Taxonomy of Breast Cancer International Consortium (METABRIC, https://ega-archive.org/), and Gene Expression Omnibus (GEO) database (https://www.ncbi.nlm.nih.gov/geo/query/acc.cgi?acc=GSE96058 accessed on 15 May 2026). A total of 1059 breast cancer patients from the TCGA database were included in the training cohort. In addition, 3069 patients from GSE96058 and 1980 patients from the METABRIC database were included in the validation cohort. Gene expression data from TCGA were obtained in transcripts per million (TPM) format. Primary tumor samples were retained, and duplicated samples were removed. Genes with near-zero variance (based on a 95% cutoff) or low expression levels (TPM < 1 in at least 70% of samples) were filtered out. Clinical information was subsequently integrated with the expression data for subsequent analysis. DDR-related genes and KLA-related genes were obtained from the Gene Set Enrichment Analysis (GSEA) database and the current literature. In this work, 384 DDRGs and 335 KLAGs were included and listed in Supplementary Table S1.

### 4.2. Identification of DEGs and Functional Enrichment Analyses

R package DESeq2 (version 1.44.0) was used for differential expression analysis. Genes with adjusted *p* value < 0.01 and |log2 fold change (FC)| ≥ 0.75 were identified as differentially expressed genes (DEGs). Gene Ontology (GO), Kyoto Encyclopedia of Genes and Genomes (KEGG) and GSEA analyses were performed to identify enriched biological processes of DEGs according to established protocols [40].

### 4.3. Construction and Validation of the KLA-DDR Gene Signature

Overall survival (OS)-related genes were screened through univariate Cox regression with *p* value < 0.05. The LASSO-Cox regression model (R package ‘glmnet’, version 4.1-8) was utilized to select the most influential model genes. According to the exported ‘lambda.min’ value, model genes were selected to compose the KLA-DDR gene signature. The KLA and DDR index (KLDRI) of each patient was calculated as the sum of each model gene’s expression level multiplied by its corresponding coefficient. The coefficients in the formula were obtained by fitting a Cox proportional hazard model with LASSO regularization. Based on the calculated risk scores, patients were stratified into high- and low-risk groups using the median risk score as the cutoff value. R package ‘survminer’ (version 0.5.0) was used to perform Kaplan–Meier analysis, and ‘timeROC’ (version 0.4) was used to present ROC curves in training and validation cohorts.

### 4.4. Tumor Microenvironment Analysis

R package ‘IOBR’ (version 0.99.0) was utilized to assess and visualize the tumor immune microenvironment through multiple algorithms, including CIBERSORT, EPIC, and quantiseq. Specifically, the correlation of the infiltration of 22 immune cells with risk score and model genes was assessed using the CIBERSORT algorithm and visualized as a heatmap. The ssGSEA algorithm (R package ‘GSVA’, version 1.52.3) was also utilized to quantify the enrichment of immune cell types in breast cancer tumor microenvironment between the high- and low-risk groups.

### 4.5. Drug Sensitivity Analysis

Drug sensitivity analysis was performed using the R package ‘oncopredict’ (version 1.2). Correlations between risk score, model genes, and the IC50 value of breast cancer-related drugs were calculated and are presented as a dot plot. The IC50 values of different drugs in the two risk groups were also calculated and visualized through boxplots. Correlation between risk score and IC50 values was also illustrated as a correlation scatter plot.

### 4.6. Establishment and Validation of the Nomogram

The prognostic nomogram was established via multivariate Cox regression, incorporating three variables: risk score, age, and stage. A decision curve analysis (DCA) plot was constructed to compare models incorporating different variables (R package ‘dcurves’, version 0.5.0). A calibration plot was used to evaluate the predictive performance of the nomogram model (R package ‘rms’, version 6.8-2).

### 4.7. Single-Cell RNA Sequencing (scRNA-Seq) Data Processing and Analysis

scRNA-seq data were obtained from the GEO database (https://www.ncbi.nlm.nih.gov/geo/query/acc.cgi, accessed on 13 May 2026) with accession code GSE176078. This dataset contains 26 primary breast cancer samples, including 5 human epidermal growth factor receptor 2 (HER2)-positive/estrogen receptor (ER)-negative, 11 ER-positive/HER2-negative, and 10 triple-negative breast cancer (TNBC) samples. R package Seurat (version 5.1.0) was used to analyze scRNA-seq data according to standard analysis procedures. Qualified cells are identified with unique molecular identifier (UMI) counts greater than 1000, detected genes more than 200 but less than 6000 and mitochondrial gene less than 20% in UMI counts. The t-SNE and uniform manifold approximation and projection (umap) methods were utilized to visualize cell clusters. Different cell types were manually annotated based on the identification of cellular molecular markers.

### 4.8. Cell Culture

Breast cancer cell lines (T47D, MDA-MB-231, MCF-10A, and SK-BR-3) were obtained from the American Type Culture Collection (ATCC). Cells were cultured in high-glucose DMEM (Servicebio, Wuhan, China, G4515-500ML) supplemented with 10% fetal bovine serum (Servicebio, Wuhan, China, G8003-100ML) and 1% Penicillin Streptomycin (Pricella, Wuhan, China, PB180120) in an incubator at 37 °C with 5% CO_2_.

### 4.9. Cell Transfection

To knock down RPA3 gene expression, small interfering RNA (siRNAs) were transfected into cells with jetPRIME according to the manufacturer’s instructions (SARTORIUS, Göttingen, Germany, jetPRIME^®^ 1.5 mL). The siRNA sequences were purchased from HeYuan Biology (Shanghai, China). The following siRNA sequences were used:

siRPA3-1: sense 5′-GGAAGUGGUUGGAAGAGUATT-3′; antisense 5′-UACUCUUCCAACCACUUCCTT-3′

siRPA3-2: sense 5′-CCAUCUUGUGUACAUCUUATT-3′; antisense 5′-UAAGAUGUACACAAGAUGGTT-3′

siRPA3-3: sense 5′-GAUUGUGCAACAUGAUUGATT-3′; antisense 5′-UCAAUCAUGUUGCACAAUCT-3′

A non-targeting siRNA was used as control: sense 5′-UUCUCCGAACGUGUCACGUTT-3′; antisense 5′-ACGUGACACGUUCGGAGAATT-3′

### 4.10. Western Blot

Western blot was carried out following established protocols [41]. Cells were lysed using RIPA buffer (Beyotime, Shanghai, China, P0013C) supplemented with protease and phosphatase inhibitors (Beyotime, Shanghai, China P1045). Equal amounts of protein were separated with SDS-PAGE (Epizyme, Cambridge, Massachusetts, USA, PG213) and transferred to PVDF membranes (MilliporeSigma, Burlington, Massachusetts, USA, Immobilon-P PVDF Transfer Membranes, 0000390118). Membranes were incubated with primary antibodies (RPA3 antibody, 1:1000, Proteintech, Wuhan, China, Cat No. 10692-1-AP; Anti-L-Lactyllysine Rabbit mAb, 1:2000, PTM Bio, PTM-1401RM) overnight at 4 °C, followed by HRP-conjugated secondary antibodies (Beyotime, Shanghai, China, A0208, A0216). Signals were detected using enhanced chemiluminescence (ECL) reagents (Abbkine, Wuhan, China, BMU102-EN).

### 4.11. RNA Extraction and qRT-PCR

RNA extraction and qRT-PCR were performed according to established protocols [42]. Total RNA was extracted using a SimplyP Total RNA Extraction Kit (BioFlux, Tokyo, Japan, BSC52M1), and cDNA was synthesized with 500 ng RNA using the HiScript II Q Select RT superMix for the qPCR kit (Vazyme, Nanjing, China, R233-01). qPCR was performed on 2 ng of cDNA samples using a Light Cycler 480 SYBR Green I Master Mix (Servicebio, Wuhan, China, G3326-05) supplemented with 0.625 μM forward primer and 0.625 μM reverse primer, and fluorescence was measured using a Light Cycler 480 II instrument (Roche, Basel, Switzerland). Relative gene expression was calculated using the 2^−ΔΔCt^ method, with HPRT1 and RPLP0 as internal references. The primers used were synthesized by Sangon Biotech, and the sequences were as follows:

RPA3: Forward: ATGGTGGACATGATGGACTTGCC,

Reverse: GTGGCCTTGGCGGTTACTCTTC.

MORF4L2: Forward: TTCTCAACCTCGTGGACAGCAATC,

Reverse: GTCCCAGTCCTCAACAAGCCATG.

PGK1: Forward: CACTGTGGCTTCTGGCATACCTG,

Reverse: GGCTGACTTTATCCTCCGTGTTCC.

RAD54B: Forward: ACCAAGTCAGTTGCAGGGGAATTC,

Reverse: ACCAGCCTGTGCCTCCTCATC.

### 4.12. CCK8, EdU, and Colony Formation Assays

MDA-MB-231 and T47D cells were seeded in a 96-well plate for CCK8 assay at a density of 3 × 10^3^ and 5 × 10^3^ cells per well, respectively, in 100 μL complete medium and then allowed to adhere overnight. For cell viability analysis, OD450 was measured every 24 h. The entire medium was replaced with 10% CCK-8 reagent (CORYEABIO, Shanghai, China, Cat#KS302) and incubated at 37 °C for 1 h. Absorbance was measured at 450 nm. For drug sensitivity analysis, cells were exposed to different concentrations of cisplatin (0, 1.56, 3.125, 6.25, 12.5, 25, 50, 100, 200, and 400μM for MDA-MB-231 and 0, 0.2, 0.4, 0.78, 1.56, 3.125, 6.25, 12.5, 25, and 50μM for T47D) for 48 h, and OD450 was measured as described above. The IC50 was calculated using GraphPad Prism (version 10.1.2).

The EdU assay was carried out according to the manufacturer’s instructions (Abbkine, Wuhan, China, KTA2030). MDA-MB-231 and T47D cells were seeded in 6-well plates at a density of 3 × 10^5^ and 5 × 10^5^ per well, respectively, and allowed to adhere overnight. Then, the cells were incubated with EdU (10μM) for 2 h, fixed with 4% paraformaldehyde (Biosharp, Hefei, China, BL539A), permeabilized with 0.5% Triton X-100 (Biofroxx, Einhausen, Germany, 1139ML500), and stained with the Click-iT reaction cocktail. Nuclei were counterstained with Hoechst, and EdU-positive cells were visualized and quantified under a fluorescence microscope.

For colony formation assay, MDA-MB-231 and T47D cells were seeded in 6-well plates (800 and 1000 cells/well, respectively) and cultured for 10–14 days. The medium was replaced every 3 days. Colonies were fixed with 4% paraformaldehyde and stained with 0.1% crystal violet (Servicebio, Wuhan, China, G1014-50ML). Colonies were counted using ImageJ software (version 1.53f51).

### 4.13. Cell Scratch Assays and Transwell Assays

For cell scratch assays, MDA-MB-231 and T47D cells were seeded in 6-well plates at a density of 6 × 10^5^ and 8 ×10^5^ per well. Cells were cultured until approximately 90–100% confluence; then, a straight scratch was made using a 200 μL pipette tip. After washing with PBS to remove debris, cells were cultured in serum-free medium. Wound closure was photographed at 0 and 24–48 h using a microscope. For transwell assays, cells were seeded into the upper chamber of transwell inserts (Corning) in serum-free medium at a density of 1 × 10^5^ per well. Medium containing 10% fetal bovine serum was added to the lower chamber. After 24–48 h of incubation, migrated cells were fixed, stained with crystal violet, and counted under a microscope.

### 4.14. Immunofluorescence Assays

For immunofluorescence assays, MDA-MB-231 and T47D cells were seeded on glass coverslips in 24-well plates at a density of 6 × 10^4^ and 8 × 10^4^, respectively. After 1–2 days of incubation, the cells were fixed with 4% paraformaldehyde for 15 min at room temperature, followed by permeabilization with 0.1% Triton X-100 for 10 min. After blocking with 5% bovine serum albumin for 1 h and washing with PBS, cells were incubated with primary antibodies (Anti-L-Lactyllysine Rabbit mAb, 1:100, PTM Bio, Hangzhou, China, PTM-1401RM) overnight at 4 °C. After washing with PBS, fluorophore-conjugated secondary antibodies (Dylight 594, Goat Anti-Rabbit IgG, Abbkine, Wuhan, China, A23420) were applied for 1 h at room temperature in the dark. Nuclei were counterstained with DAPI, and images were captured using a fluorescence microscope under identical exposure settings.

### 4.15. Statistical Analysis

All statistical analyses were performed with R software (V. 4.4.0). Student’s *t*-test (for normally distributed data) or the Wilcoxon test (for non-normally distributed data) was used to analyze differences between the two groups. Comparisons between multiple groups were analyzed using the Kruskal–Wallis test. Survival curves were described with Kaplan–Meier curves and compared with the log-rank test. Hazard ratios (HRs) and 95% confidence intervals (CIs) were calculated using uni- and multivariate Cox proportional hazards regression models. *p* < 0.05 was considered to be statistically significant. For DEG and functional enrichment analyses, multiple testing correction was performed using the Benjamini–Hochberg (BH) method. Adjusted *p*-values < 0.05 were considered statistically significant.

## 5. Conclusions

In conclusion, we established the KLDRI signature based on KLAGs and DDRGs in breast cancer patients. The KLDRI signature can be utilized to well stratify patients into different risk groups and predict their prognosis with consistent performance in multiple datasets. The KLDRI signature has the potential to guide the individualized and precise diagnosis and treatment of patients with breast cancer. We also demonstrated the oncogenic role of RPA3, which could serve as a potential biomarker and candidate therapeutic target.

## Data Availability

Both RNA sequencing and single-cell sequencing are available in public databases, including The Cancer Genome Atlas (TCGA, https://portal.gdc.cancer.gov/), Molecular Taxonomy of Breast Cancer International Consortium (METABRIC, https://ega-archive.org/) and the Gene Expression Omnibus (GEO) database (https://www.ncbi.nlm.nih.gov/geo/).

## Supplementary Materials

The following supporting information can be downloaded at https://www.mdpi.com/article/10.3390/ijms27104493/s1.

## Abbreviations

The following abbreviations are used in this manuscript:

CTRP	Cancer Therapeutics Response Portal
DCA	Decision Curve Analysis
DDR	DNA Damage Repair
DEGs	Differentially Expressed Genes
ER	Estrogen Receptor
GDSC	Genomics of Drug Sensitivity in Cancer
GEO	Gene Expression Omnibus
GO	Gene Ontology
GSEA	Gene Set Enrichment Analysis
HER2	Human Epidermal Growth Factor Receptor 2
HR	Hazard Ratio
IC50	Half Maximal Inhibitory Concentration
KEGG	Kyoto Encyclopedia of Genes and Genomes
KLA	Lysine Lactylation
METABRIC	Molecular Taxonomy of Breast Cancer International Consortium
OS	Overall Survival
ROC	Receiver Operating Characteristic
SNV	Single Nucleotide Variant
TCGA	The Cancer Genome Atlas
TNBC	Triple-Negative Breast Cancer
t-SNE	t-distributed Stochastic Neighbor Embedding
UMI	Unique Molecular Identifier
EdU	5-ethynyl-2′-deoxyuridine
