# Supplementary material for: Construction of a Lysine Lactylation- and DNA Damage Repair-Related Gene Signature to Predict the Prognosis and Drug Sensitivity of Breast Cancer Patients
Source: Int J Mol Sci. 2026 May 17;27(10):4493. doi: 10.3390/ijms27104493 (PMC13207498; doi:10.3390/ijms27104493)
Supplement: Supplementary file 1 [file ijms-27-04493-s001.zip › Supplementary Figures.pdf]

Supplementary Figure S1

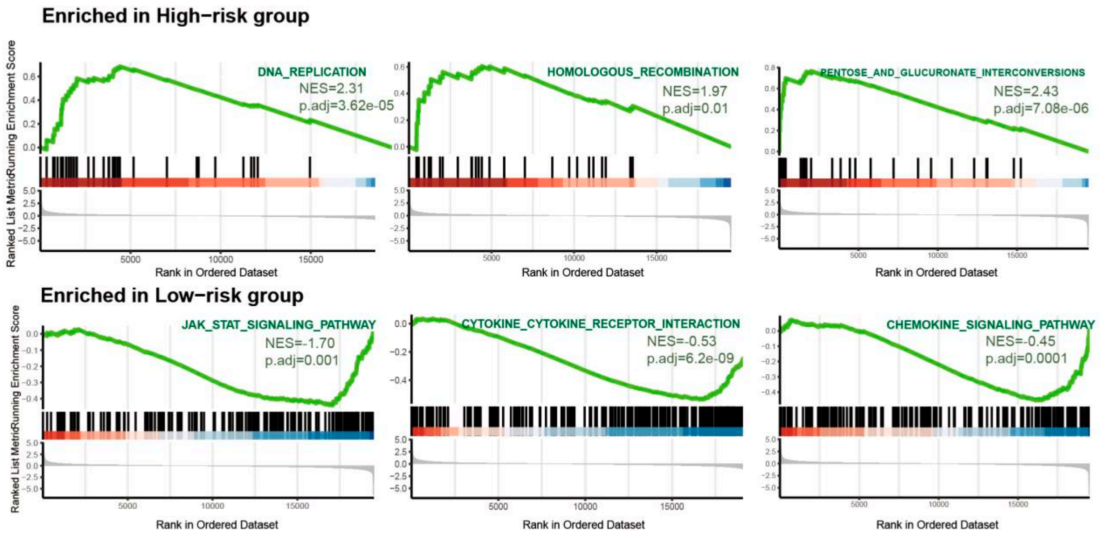

Supplementary Figure S1. GSEA enrichment analysis of DEGs. The running enrichment score curve reaches its peak on the left or right side of the ranked gene list, indicating that genes in the corresponding pathway are predominantly enriched in the high-risk group (left) or low-risk group (right)

Supplementary Figure S2

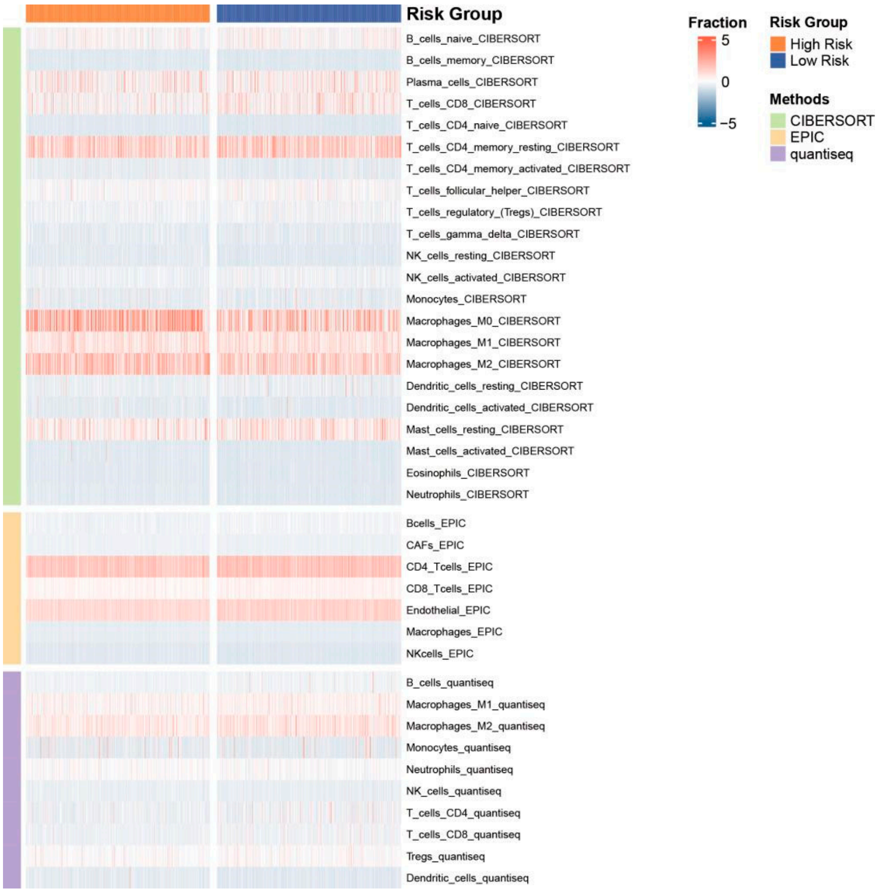

Supplementary Figure S2. Immune infiltration status between different risk groups analyzed using the CIBERSORT, EPIC and quanTIseq algorithms.

Supplementary Figure S3

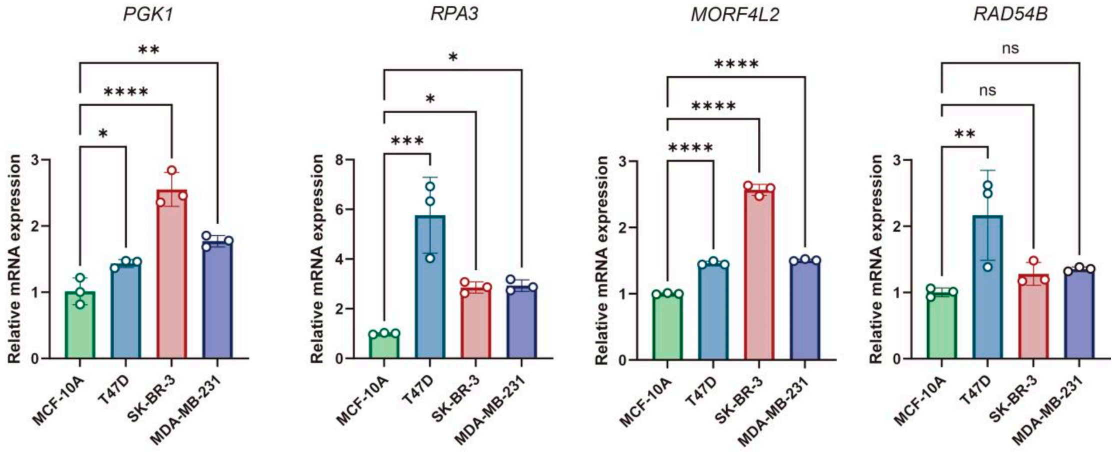

Supplementary Figure S3. Relative mRNA expression levels of *PGK1, RPA3, MORF4L2* and *RAD54B* in MCF-10A, T47D, SK-BR-3 and MDA-MB-231 cell lines.
